# Honor and Eugenics

**Published:** 1915-05

**Authors:** 


### The Department of Eugenics, Mental and Social Hygiene

CONDUCTED BY
DR. MALONE DUGGAN
813-814 Gibbs Building, San Antonio, Texas
All communications for this department should be sent to DR. DUGGAN

### Honor and Eugenics.

A strange subject you think. It is not as strange as it seems. On closer investigation you will find that honor is the switchboard to the development of every character; the test of the character of every development. Heredity and environment are but the handmaidens of eugenics; their efficiency depending upon the quality of the character and the opportunities they afford. This quality is determined absolutely by the sense of honor practiced in the development of the environment. Therefore, the importance of the tone power of honor. Honor suggests right living and right relations toward others. It requires, unalterable, self-interest to be consistent with other interest. And, therefore, to secure self-interest, the interest of others must be regarded first. This is the foundation of ethics and insures our eternal progress.

However, before a quality can be felt as an influence on others, it must first become a part of the deeper self. When so sublimed it will radiate as an actual power and produce personal attractions. In this wise honor can influence and direct the character of the man; determining his personality and influence in society. It can banish fear, worry and suspicion. It can inspire courage, hope, and stimulate the mind, creating faith in others and developing personal freedom. There is no sense of satisfaction so great as the feeling of doing no wrong and the consciousness of having done some good. If the race is ever to be regenerated it will come through the greater appreciation of the element of personal honor. Reflect for a moment on the significance of this influence on man himself and his environment; the benefit to be derived from its practice in securing the best conditions possible for his development. When all best interests are respected the negative elements of selfishness and deceit are rooted out and the growth of body and freedom of mind flourishes as the green bay tree; a finer sense of judgment and intelligence is thereby displayed and the consequent result is a continuous and harmonious evolution.

